# Application of records theory on the COVID-19 pandemic in Lebanon: prediction and prevention

**DOI:** 10.1017/S0950268820001909

**Published:** 2020-08-26

**Authors:** Zaher Khraibani, Jinane Khraibani, Marwan Kobeissi, Alya Atoui

**Affiliations:** 1 Department of Applied Mathematics, Faculty of Sciences, Lebanese University, Hadath, Lebanon; 2 Rammal Rammal Laboratory, Physio-toxicité Environmental (PhyToxE) Research Group, Faculty of Sciences, Lebanese University, Nabatieh, Lebanon; 3 Division of Infectious Diseases, Sahel General Hospital, Beirut, Lebanon; 4 Rammal Rammal Laboratory, Applied organic synthesis Research Group (SOA), Faculty of Sciences, Lebanese University, Nabatieh, Lebanon; 5 Laboratoire Eau, Environnement et Systèmes Urbains (LEESU), Université Paris Est-France, Champs-sur-Marne, France

**Keywords:** COVID-19, emerging infectious diseases, Lebanon, non-parametric test, pandemic, prediction, records theory, sporadic

## Abstract

Given the fast spread of the novel coronavirus (COVID-19) worldwide and its classification by the World Health Organization (WHO) as being one of the worst pandemics in history, several scientific studies are carried out using various statistical and mathematical models to predict and study the likely evolution of this pandemic in the world. In the present research paper, we present a brief study aiming to predict the probability of reaching a new record number of COVID-19 cases in Lebanon, based on the record theory, giving more insights about the rate of its quick spread in Lebanon. The main advantage of the records theory resides in avoiding several statistical constraints concerning the choice of the underlying distribution and the quality of the residuals. In addition, this theory could be used, in cases where the number of available observations is somehow small. Moreover, this theory offers an alternative solution in case where machine learning techniques and long-term memory models are inapplicable because they need a considerable amount of data to be performant. The originality of this paper lies in presenting a new statistical approach allowing the early detection of unexpected phenomena such as the new pandemic COVID-19. For this purpose, we used epidemiological data from Johns Hopkins University to predict the trend of COVID-2019 in Lebanon. Our method is useful in calculating the probability of reaching a new record as well as studying the propagation of the disease. It also computes the probabilities of the waiting time to observe the future COVID-19 record. Our results obviously confirm the quick spread of the disease in Lebanon over a short time.

## Introduction

The evolution in the diversity of human and animal pathologies has been favoured through time by a continuously increasing number of factors. Migrations, travel and trade, abuse in medical or veterinary practices (antibiotics prescriptions, drugs for humans made from animal products) are among the major factors. Others could be added to the list such as climate changes and the risks of bioterrorism acts.

In this highly evolving context, the early assessment of an emerging risk of a disease in a given population, as well as the epidemiological survey are powerful tools for efficient controlled prevention. Important parameters such as the incubation time, contagion process, transmissibility and more specifically spread, are of crucial priority to predict. This allows the quick gain of the necessary knowledge for the medical community to act in an ordered and predictable environment. The good prediction of these key parameters allows, in general, acceptable management of a disease by the concerned authorities. It also permits the enhancement of the capabilities of a given nation to face unexpected dangerous pandemics with high control, order, and success. As a consequence, the battle against the disease could be softer in terms of fatalities and the economy. Good initial evaluation requires a suitable surveillance system for diagnosis methods. In parallel, available statistical methods and models are highly recommended for computing the risk of amplification of the disease. This is done directly from the first detected cases regardless of the total final magnitude in time or size and the real cause of this magnitude (direct or indirect transmissibility of the disease). Therefore, facing the occurrence of a new phenomenon as for the COVID-19 is highly advised to verify whether it is sporadic or not. Specific precautions should be taken at the onset of an outbreak.

In this paper, we would like to investigate the novel coronavirus (COVID-19) in a statistical approach. This virus belongs to a large family of viruses that may cause severe ill cases in humans or animals. The first cases of COVID-19 were announced in December 2019 in Wuhan, China.

The new virus targets the lower part in the respiratory system causing mild to severe cases of lung infections (pneumonia), thus requiring a special treatment. In contrast to its predecessors from the same family, the challenge with this novel coronavirus is its enigmatic approach of spreading in human. It spreads primarily through droplets of saliva or discharge from the mouth or nose when an infected person coughs or sneezes.

Indirectly, it could be transmitted from contact with contaminated surfaces or objects and then touching critical parts of the body such as the mouth, nose, eyes and so on [1].

The first known case of COVID-19 was revealed in China and the disease began to spread among people to become a global pandemic in a short period.

Infected people from different regions in the world entered Lebanon and transmitted the virus to Lebanese individuals. In a short while, the Lebanese authorities found the infection within their borders. The number of cases increased rapidly, and only within a few days, Lebanon itself became an infected area.

At the time of writing this paper, several studies had been conducted using various statistical and mathematical models to predict the probable evolution of this epidemic in the world [2–7]. Herein, we propose a new simple probabilistic method based on the record theory. This method proved to be interesting for the accurate calculation of the probability of reaching a new record as well as studying the propagation of COVID-19.

The records theory has been successfully applied in several scientific areas like hydrology, meteorology, epidemiology, sports and natural phenomena (see for details [8–14]). In this study, we used a non-parametric test developed by Khraibani *et al*. in 2015 [8] and based on the number of records for the early detection of emerging events.

We consider *S*_1_ < ⋅ ⋅ ⋅ < *S*_*n*_ be the first *n* independent occurrence of a new event and let {Δ*S*_*n*_: *n* ≥ 1} be the sequence of real random variables that represent the waiting time between two successive events. In the context of random variables independent and identically distributed (i.i.d.) {Δ*S*_*n*_}, the emergence is characterised by the smallest Δ*S*_*n*_ (*S*_*n*_, *S*_*n*−1_ are very close together) and the test statistic used is the process of lower records (Δ*S*_*n*_)^−1^.

The authors propose for the study a robust and exact non-parametric test statistic against exponential growth to detect an emerging phenomenon. Their proposed statistic *N*_*n*_ is based on the number of observed records and an important characteristic of *N*_*n*_, the probability distribution being independent of the observations. Moreover, they consider *H*_0_:ρ = 1 (the event remains sporadic) and *H*_1_:ρ > 1 (the event is an emerging new phenomenon), and they assume that Δ*S*_*n*_ has an exponential distribution with an unknown parameter λ > 0. In the case where ρ > 1 they assume that there is an exponential growth in *S*_1_, *S*_2_, …, *S*_*n*_, which are independent continuous random variables but not necessarily identically distributed. In case of ρ = 1, *S*_1_, *S*_2_, …, *S*_*n*_ are independent and identical (i.i.d.). It is of importance to note that ρ denotes the ‘constant rate of the exponential growth of the sequence’ [8]. The records theory is quite advanced for i.i.d. random variables with continuous cumulative distribution function (CDF). In our setting, the interest of the records theory from the inference point of view consists of the robustness of the distribution of the indices of records and the distribution of the number of records since these distributions may be exactly calculated and are moreover independent of under *H*_0_ and *H*_1_. Therefore, it is particularly interesting for the early detection of an emergent phenomenon based on a small number of observations. It is also to be underlined that record theory focuses on the records values and records times of extreme events, a characteristic that enriches the analysis of potential results. Record times are taken into account through specific random variables called record indicators [15]. We apply here the records theory on the daily prevalence data of the unprecedented COVID-19 in Lebanon from the 21st of February 2020 until the 13th of March 2020. The data were collected from the official website of Johns Hopkins University [16].

## Methods

### Records sequences and notation

Let (Ω, *F*, *P*) be a probability space. A random variable *X* is an *F*-measurable function *X*:Ω →  ℝ. An observation *X*_*n*_ is called an upper record noted *R*_*n*_ if it is at least as large as the maximum of all preceding observations: *X*_*n*_ > max {*X*_1_, …, *X*_*n*−1_}. Note that *R*_1_ = *X*_1_ trivial record. We also define δ_*n*_ as a record indicator variable of the *n*th observation, that is δ_1_ = 1 (the first observation is a trivial record), and, for *n* > 1,
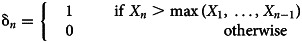


The record times *L*_*n*_ is defined by:
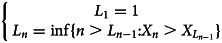


With these notations, 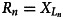.

We define the record counting process or the number of records among {*X*_1_, …, *X*_*n*_} including the trivial record *X*_1_ as
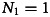

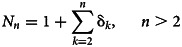


### Mathematical definition of an emerging disease

The starting event is the transition between the stability state ‘0 pathogen’ (or of the state ‘equilibrium of the ecosystem of pathogens’) and the instability of this state.

**Fig. 1.**
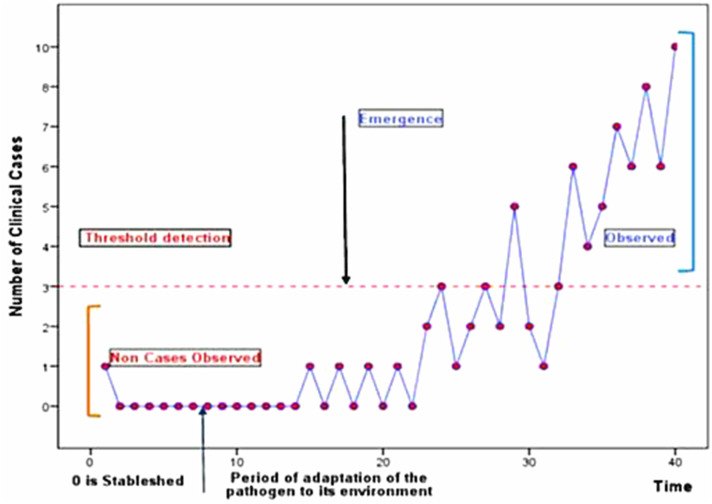
Emergence of a disease.

The emerging process concerns the consequences of this instability over a sufficient period to become visible. It thus needs the instability to perdure with the existence of a minimum threshold over which the population of pathogens is directly or indirectly noticed and seen (Fig. 1).

Let *Y*_*n*_ be the percentage of clinical cases in a population at the time *n*, such as *Y*_*n*_ =  *f*(*Y*_*n*−1_) and *f*(0) = 0. By using the Taylor's first-order series approximation near 0 and by supposing *Y*_0_ small:
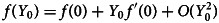

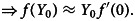

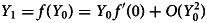


Thus
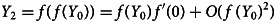

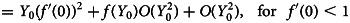


In case of a non-emerging process (*H*_0_):
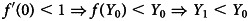


But, *f*(*Y*_1_) = *f*(*f*(*Y*_0_)) ≈ *f*(*Y*_0_)*f*′(0) < *Y*_0_. We obtain by recurrence that *Y*_*n*_ : =*f*^(*n*)^(*Y*_0_) remains of same order of magnitude as *Y*_0_.

In case of an emerging process *H*_1_: *f*(0) >  1 and we obtain:

*f*(*Y*_0_) > *Y*_0_ ↠ *Y*_1_ > *Y*_0_ thus, *Y*_*n*_ will no more remain negligible.

**Fig. 2.**
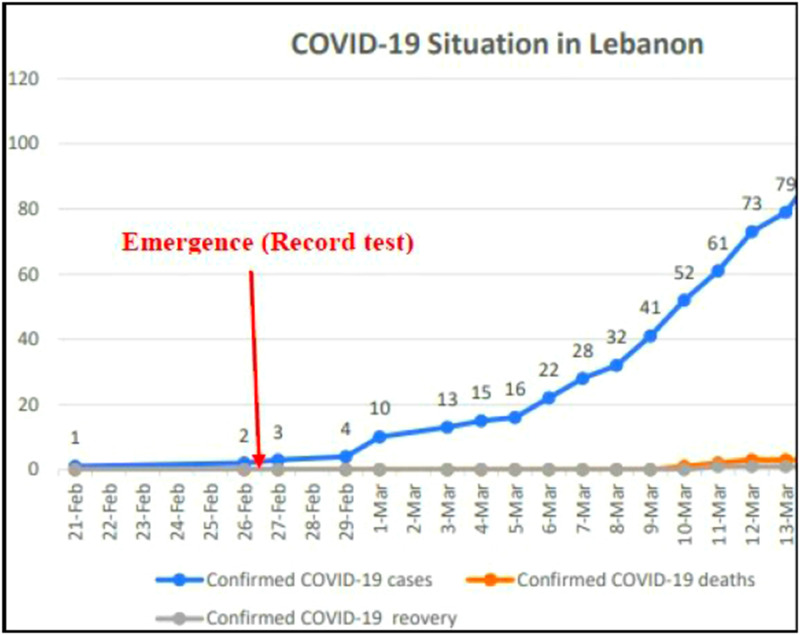
Daily cumulative/emerged number of confirmed, fatal and recovered cases of Coronavirus Disease 2019 (COVID-19) in Lebanon. *Data source*: MOPH, 2020.

We give some descriptive about the COVID-19 pandemic in Lebanon from the birth date of the virus (see Fig. 2). By theory, the true number of individuals with COVID-19 infection cannot be accurately determined regardless of the detection measures. As a consequence, we observed a few cases at the beginning of COVID-19, between the 21st and 29th of February, with no background information about the future evolution of the pandemic. In the following, we use the record test to detect the emergence of COVID-19 from a small number of observations recorded between the 21st of February and the 13th of March 2020 (see Figs. 1 and 2).

### Test description

We select here the robust statistic *N*_*n*_ as a statistic of test of *H*_0_. In the case of sporadic events, we assume that the waiting times between two successive cases {Δ*T*_*k*_} are i.i.d. with a common CDF. Then *H*_0_ is defined as
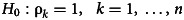


In this paper we perform a brief survey to gain further information about the degree of sporadicity of COVID-19 and the frequency of its increase. Our results are the outcome of a non-parametric test based on the number of records observed over time.

We present the principal ideas of the records test; if the new event (COVID-19) is an emerging event, then the waiting times {Δ*T*_*k*_}_{1≤*k*≤*n*}_ between two successive events should decrease. On the other hand, for a sporadic event, the {Δ*T*_*k*_}_{1≤*k*≤*n*}_ should be i.i.d. This means that *N*_*n*_, the number of lower records in the sequence; {Δ*T*_*k*_}_{1≤*k*≤*n*}_ should show tendency to increase.

Based on this idea, Khraibani *et al*. [8] assumed that the waiting times are independent for each *k* with a continuous CDF denoted *G*_*k*_. In the case of sporadic events, *G*_*k*_ is independent of *k* and the null hypothesis *H*_0_:ρ_*k*_ = 1, *k* = 1, …, *n* is adopted. While in the case of emergent, *G*_*k*_ increases with *k*. In this regard, we define the alternative hypothesis *H*_1_:ρ_*k*_ > 1. More particularly, we assume that {*G*_*k*_} belongs to the distribution family in the form 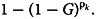


### Probability distribution of the number of record events

In this section, we give the exact probability distribution of *N*_*n*_. David and Barton [17] were pioneers in introducing an expression for the exact probability distribution. Recall that the distribution of *N*_*n*_ is independent of the observations and it can be calculated exactly for any *n* value.

Another importance of these statistics is the ease and ability to observe some records for small *n* values. The random variables δ_1_, …, δ_*n*_ are independent with probabilities of success [10]:
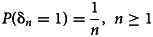


Then, the exact distribution of the number of records is independent of *F* and can be expressed compactly using Stirling numbers of the first kind 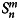:1
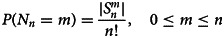


Based on the asymptotic formula of the Stirling numbers of the first kind, we deduce the asymptotic distribution of *N*_*n*_ for large sample sizes [18]:
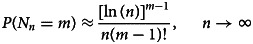


The computation of the *P*(*N*_*n*_ ≥  *m*) in the i.i.d. case (*H*_0_) are listed later.

Nevzorov [12] gives the probability of the record indicators and the number of records in the case of variables being non-independent and identically distributed (*H*_1_):
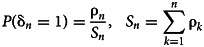
and2
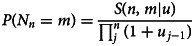


*u*: = (*u*_0_, *u*_1_, …, *u*_*n*−1_), *s*(*n*, *m*|*u*)being a generalised Stirling number of the first kind. We have *u*_*j*_ = (*a*^*j*^ − 1)(*a* − 1)^−1^*a*^−*j*^, 1 ≤  *j* ≤  *n* − 1.

In the following, we consider the alternative hypothesis *H*_1_:ρ_*k*_ = *a*^*k*^, *k* =  1, …, *n* where *a* ≥ 1, consistent with the exponential growth of an emerging phenomenon such as an infectious disease, when *a* > 1, and with sporadic events, when *a* = 1.

## Results

### Illustration of the records test on COVID-19

We applied the records of COVID-19 cases in Lebanon from the date of identification of the index case until the 13th of March 2020. The first case was announced on the 21st of February 2020. The collected data consist of the birth date and the notification date of each of the COVID-19 cases identified in Lebanon during this period. Therefore, *X*_*n*_: = (Δ*T*_*n*_)^−1^, where Δ*T*_*n*_ is the *n*th waiting time between two successive notification (resp. birth) date. We list in Table 1 our collected data.

**Table 1.** Waiting times between two successive cases and number of COVID-19 cases in Lebanon per day

*T*_*n*_	21/02	26/02	29/02	01/03	02/03	05/03	06/03	08/03	10/03	11/03	13/03
COVID	1	1	2	6	3	3	6	10	9	20	16
(Δ*T*_*n*_)	5	3	1	1	3	1	2	2	1	2	–
(Δ*T*_*n*_)^−1^	0.2	0.33	1	1	0.33	1	0.5	0.5	1	0.5	–

As shown in Table 1, the number of observed records is equal to 6 (*N*_*n*_ = 6), the record values: *R*_1_ = 0.2, *R*_2_ = 0.33, *R*_3_ = 1, *R*_4_ = 1, *R*_5_ = 1, *R*_6_ = 1. The records times: *L*_1_ = 1 (21/02), *L*_2_ = 2(26/02), *L*_3_ = 3(29/02),  *L*_4_ = 4(01/02),  *L*_5_ = 6(05/03), *L*_6_ = 9 (03/10).

From the figures presented above, we see the trajectory of {*X*_*n*_}_*n*≤10_ with the successive observed records, drawn as red circles and the number of maximal COVID-19 records between 21 February 2020 and 13 March 2020.

From equation (1), we compute under *H*_0_ the probability to observe at least *m* records among *n* variables for any *m* ≤ *n*.

According to Table 2, the probability to observe at least *m* records among n variables increases with *n*. This table is used to compute the significance level of the test of *H*_0_ : α = *P*(reject  the  sporadic  hypothesis). We decided then to apply the results of Khraibani *et al*. [8] to investigate if the incidence rate of COVID-19 in Lebanon was increasing. The test statistic of interest is thus the number of lower records in the sequence *X*_*n*_ = (Δ*T*_*n*_)^−1^. The records test of *H*_0_ depends on the number of observed records *N*_*n*_ and the notification dates of occurrence.

**Table 2.** *P*(*N*_*n*_ ≥  *m*), for *n* = 10, 20 and for different values of *m*

	*m*						
*n*	1	2	3	4	5	6	7
10	1	0.9000	0.6171	0.2939	0.0945	0.0203	0.0029
20	1	0.9500	0.7726	0.4978	0.2470	0.0944	0.0280

**Fig. 3.**
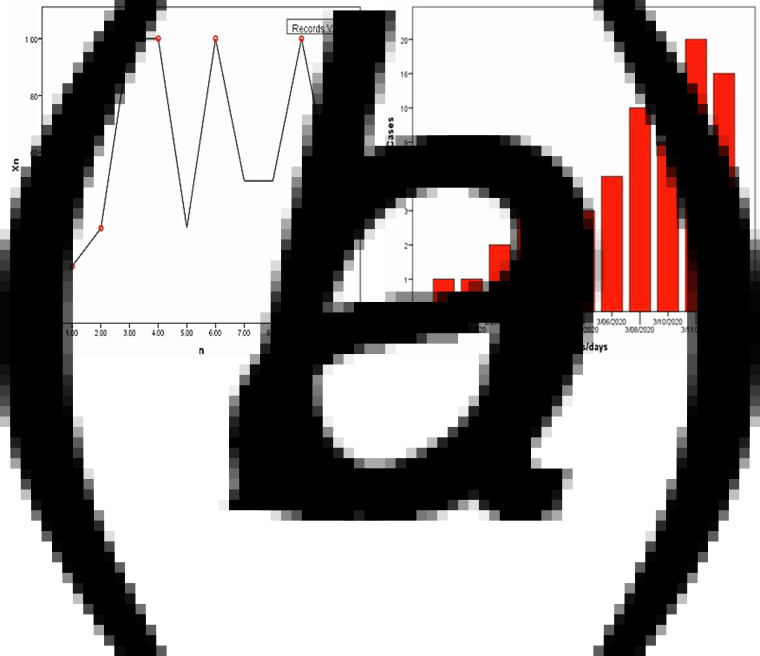
(a) Records values of *X*_*n*_ = (Δ*T*_*n*_)^−1^. (b) Number of observed COVID-19 cases per day.

We observe five records at discrete times *n*  =  1, 2, 3, 4, 6 (Fig. 3a). We have *P*(*N*_10_ ≥ 6) = 0.0203 (see Table 2), allowing one to reject *H*_0_ with a small error probability. From only a few reported cases, we conclude that the COVID-19 is an emerging disease in Lebanon. Also, from equation (1) and Table 2, the calculated 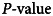 for the hypothesis *H*_0_ confirm that COVID-19 in Lebanon represents an increasing phenomenon. The *H*_0_ is then rejected, so that to calculate under *H*_1_ the probability of the numbers of records by using equation (2).

Khraibani *et al*. [8] considered two values of *a*: *a* = 1.1 and *a* = 1.5, the first, corresponding to a slow emergence, and the second one, is characteristic of a quick emergence.

Based on these values of *a*, we can easily compute under *H*_1_ the probability of records number, exceeding the records values *m* = 6, 7, 8, 9 for different values of *n* = 10, 20, 30 days:

From the results of Table 3, one could notice the COVID-19 in Lebanon emerges in a very rapidly with a high probability of exceeding some record values.

**Table 3.** *P*(*N*_*n*_ ≥  *m*) under *H*_1_, for *n* = 10, 20, 30 and for different values of *m* and *a*

*N*	10	20	30
*m*	6	7	8
*P*(*N*_*n*_ ≥ *m*), *a* = 1.1	0.0455	0.1108	0.14261
*P*(*N*_*n*_ ≥ *m*), *a* = 1.5	0.2683	0.7554	0.9352

By taking into account the future COVID-19 records, we compute the probabilities of waiting time to observe a new record 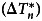 by using the following equation:3
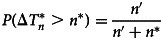


Consequently, the probabilities of observing a COVID-19 record in Lebanon for the next 5 days can be easily calculated from equation (3). For example the number of days in the database, *n*′ = 22 days (between 21 February 2020 and 13 March 2020), we deduce the probability of waiting time for a new record: 22/(22 + 5) ≈ 0.82; confirming the rapid growth of COVID-19 in Lebanon over a short period.

The fact that an event occurring for the first time becomes a frequently encountered phenomenon manifests itself by shortening of inter-event times which corresponds to an increasing trend in the series of observations {*X*_1_, …, *X*_*n*_}. From all these observations, we conclude that our test efficiently proves that COVID-19 in Lebanon emerges very quickly resulting in a high probability of exceeding some observed record values and waiting time. The early detection of the risk of quick propagation of COVID-19 in Lebanon is of great value since it could increase the chances for adequate prevention. The existence of a good surveillance system and epidemiological surveys help in preventing the complete failure of even the most well-developed public healthcare systems in the world.

## Conclusion

Facing the first time of occurrence of cases of a new disease, such as COVID-19, it is crucial to predict the degree of sporadicity or emergence of the pandemic. We propose in this paper a non-parametric exact test for the early detection of emerging events based on the number of lower records *N*_*n*_ in *X*_*n*_; which *X*_*n*_ = (Δ*T*_*n*_)^−1^ the waiting time between two successive COVID-19 cases. The method presented in this paper is a general method that could be applied for new diseases with no epidemiological information. It is a robust tool allowing the calculation of exact distribution even for small size samples. We were able, using this method, to detect the quick propagation of COVID-19 in Lebanon from a small number of cases. First, we consider the hypothesis *H*_0_:ρ_*k*_ = 1 (sporadic cases), and we assume that inter-event times have an exponential distribution with unknown parameter λ > 0; then we suppose the alternative hypothesis *H*_1_:ρ_*k*_ > 1; with ρ_*k*_ = *a*^*k*^ and for *a* > 1, we obtain an exponential increase in the occurrence concentrations of the developing phenomenon.

In summary, we confirm using our test that COVID-19 is spreading very quickly in Lebanon. For the future research, we recommend better epidemiological surveillance of epidemics in order to minimise the risks of their transformation into pandemics. This prevention could preserve the health care systems of even strong countries from crash. Several other statistical methods are also to be tested in the follow-up of COVID-19 especially in Lebanon. This work is in due course in our lab.

## Data Availability

Requests for access to the data that support this study should be made to the corresponding author, Z. Khraibani.

## References

[1] World Health Organization. WHO characterizes COVID-19 as a pandemic. https://www.who.int/emergencies/diseases/novel-coronavirus-2019 Accessed 13 March 2020.]

[2] Benvenuto D (2020) Application of the ARIMA model on the COVID-2019 epidemic dataset. Data in Brief 29, 1–4. doi: 10.1016/j.dib.2020.105340. PMC7063124 32181302

[3] Qianying L (2020) A conceptual model for the coronavirus disease 2019 (COVID-19) outbreak in Wuhan, China with individual reaction and governmental action. International Journal of Infectious Diseases 93, 211–216. 32145465 10.1016/j.ijid.2020.02.058 PMC7102659

[4] Kucharski A (2020) Early dynamics of transmission and control of 2019-nCoV: a mathematical modelling study. Lancet Infectious Diseases 20, 553–558. 32171059 10.1016/S1473-3099(20)30144-4 PMC7158569

[5] Siettos L (2013) Mathematical modeling of infectious disease dynamics. Virulence 4, 295–306. 23552814 10.4161/viru.24041 PMC3710332

[6] Jonathan M (2020) Novel coronavirus 2019-nCoV: early estimation of epidemiological parameters and epidemic predictions. medRxiv. doi: 10.1101/2020.01.23.20018549. PMC8165596 34053269

[7] Haidar N (2020) Passengers destinations from China: low risk of Novel Coronavirus (2019-nCoV) transmission into Africa and South America. Epidemiology and Infection 148, e41. Published 2020 February 26. 32100667 10.1017/S0950268820000424 PMC7058650

[8] Khraibani Z (2015) A non parametric exact test based on the number of records for an early detection of emerging events: illustration in epidemiology. Communications in Statistics-Theory and Methods 44, 726–749.

[9] Ahsanullah M (2004) Record Values-Theory and Applications. Oxford: University Press of America.

[10] Arnold B (1998) Records. New York: John Wiley.

[11] Khraibani Z (2011) Record method for the natural disasters application to the storm. Journal of Environmental Science and Engineering 5, 643–651.

[12] Nevzorov V (1988) Records. Theory of Probability & Its Applications 32, 201–228.

[13] Nevzorov V (2000) Records: Mathematical Theory. Province, RI 02940, USA. Lanham, MD: American Mathematical Society.

[14] Gulati S (2019) Analysis of hurricane extremes and record values in the Atlantic. Communications in statistics: case studies. Data Analysis and Applications 5, 101–110.

[15] Rocco B (1987) Embedding sequences of successive maxima in extremal processes, with applications. Journal of Applied Probability 24, 827–837.

[16] Official website of Johns Hopkins University. [Online] [Cited: March 13, 2020.]. Available at https://gisanddata.maps.arcgis.com/apps/.

[17] David F (1962) Combinatorial Chance. New York: Hafner Publishing Co, p. 356.

[18] Renyi A (1962) On the extreme element of observations. Oszt Kazl 2, 105–121.

